# Quality attributes of the developed banana flour: Effects of drying methods

**DOI:** 10.1016/j.heliyon.2023.e18312

**Published:** 2023-07-16

**Authors:** Mahfujul Alam, Mrityunjoy Biswas, Mir Meahadi Hasan, Md Faruk Hossain, Md Ashrafuzzaman Zahid, Md Sajib Al-Reza, Tarikul Islam

**Affiliations:** a Department of Agro Product Processing Technology, Jashore University of Science and Technology, Jashore, 7408, Bangladesh; b Department of Nutrition and Food Technology, Jashore University of Science and Technology, Jashore, 7408, Bangladesh; c Department of Food Technology and Nutritional Science, Mawlana Bhashani Science and Technology University, Santosh, Tangail, 1902, Bangladesh; d Department of Textile Engineering, Jashore University of Science and Technology, Jashore, 7408, Bangladesh

**Keywords:** Banana flour, Drying methods, Functional property, Bioactive compounds, Sensory attributes, Microstructures

## Abstract

The study aims to investigate the effects of different drying methods on the changes in functional properties, physicochemical composition, bioactive compounds, antioxidant activity, sensory attributes, and microstructural quality of the banana flours. Two local banana cultivars, Mehersagar and Sabri, were dried to produce flour using four distinct drying methods: freeze drying (FD), cabinet drying (CD), microwave oven drying (MOD), and forced air oven drying (FOD). The functional properties of the developed banana flours were observed where the findings were as water holding capacity (0.93 ± 0.06–2.74 ± 0.04 g water/g dry sample), oil absorption capacity (0.87 ± 0.06–2.22 ± 0.10 g oil/g dry sample), swelling capacity (4.62 ± 0.02–5.05 ± 0.03 g paste/g dry sample), bulk density (0.54 ± 0.04–0.81 ± 0.02 g/ml), tapped density (0.62 ± 0.04–0.93 ± 0.03 g/ml) and Carr's Index (9.38 ± 0.47–13.58 ± 0.43%). Freeze-dried Mehersagar cultivar's flour showed the leading functional properties with good flowability and cohesiveness. The physicochemical parameters of the flours also revealed significant differences (p < 0.05) in lightness (L*) (50.51 ± 1.49–72.21 ± 1.05), moisture content (3.96 ± 0.09–7.74 ± 0.13%), protein (2.72 ± 0.07–3.93 ± 0.06%), crude fat (0.11 ± 0.01–0.36 ± 0.04%), crude fiber (0.64 ± 0.03–1.22 ± 0.03%), carbohydrate (84.15 ± 0.24–88.26 ± 0.15%) and energy content (354.25 ± 0.57–370.02 ± 0.39 kcal/g). Total flavonoid content (21.44 ± 0.04–34.34 ± 0.03 mgQE/100g) and phenolic content (29.91 ± 0.01–71.46 ± 0.03 mgGAE/100g) was observed, while the highest retention of bioactive compounds was exhibited in Mehersagar cultivar's flour. In terms of appearance, fineness, taste, flavor, color, and overall acceptability, the dried banana flour of both the cultivars obtained from freeze-dried scored overall acceptability 8.04 ± 0.02 and 7.92 ± 0.17, respectively. The scanning electron microscopy analysis of the microstructure of flour granules from each sample revealed a diverse morphological configuration in particle size and shape. According to the findings of this study, the freeze-drying technology is superior to others, and the Mehersagar banana cultivar is more satisfactory in terms of quality characteristics. Moreover, the quality parameters of banana flour may facilitate the formulation of different flour-based gluten-free baked products and food supplements.

## Highlights

• Banana pulp flour was developed by applying different drying methods.
• Among the drying methods, freeze drying yielded the best quality banana flour.
• Functional, nutritional, and sensory qualities were restored by freeze-drying.
• High-quality banana flour was obtained from freeze-dried Mehersagar cultivar.

## Graphical abstract

**Figure:**
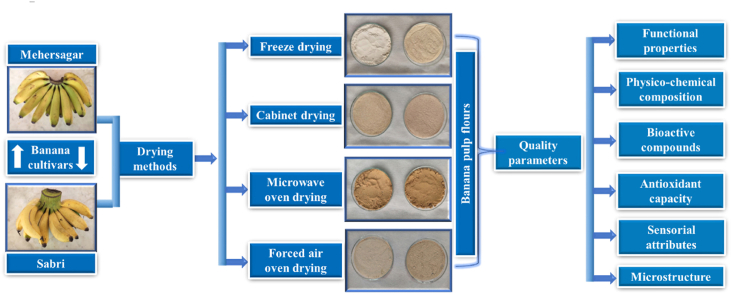

## 1 Introduction

In Bangladesh, the banana is one of the essential fruits from an herbaceous blooming plant. It is famous as healthy dessert fruit when ripened and accessible year-round, with the most excellent consumption rate of any fruit [1]. This is an economic crop among farmers due to its wide cultivation and large yearly production [2]. In Bangladesh, total banana production in 2020–2021, was recorded at 8,26,151 metric tons from the cultivated areas of about 49,449 ha [3]. There are different kinds of banana cultivars available in Bangladesh. However, banana varieties like *Musa acuminata* and *Musa paradisica* are most common and widely grown in the northern and western areas of Bangladesh [4]. Mehersagar and Sabri are both cultivars of the same *Musa acuminate* species, native to Southeast Asia, cultivated commercially in Bangladesh [5]. Banana is prominent for their viscous, sticky pulp, crimson index, and thick peel, contributing to their attractiveness [6]. Banana pulp has been examined from several perspectives for its potential to extract and separate multiple health-beneficial components, including various types of starch, cellulose, and bioactive chemicals [7]. Banana contains a great diversity of high-value bioactive compounds, such as phenolics, flavonoids, carotenoids, biogenic amines, and phytosterols, which are highly desirable in the diet due to their positive effects on human health and well-being [7]. Ripe bananas are also high in dietary fiber, resistant starch, certain vitamins (such as ascorbic acid, vitamin A, malic acid, succinic acid, palmitic acids, and folic acid), and numerous minerals (such as potassium, magnesium, phosphorus, iron, and calcium), but are naturally low in fat and sodium [8]. Due to inappropriate postharvest handling and processing, 24.62% of postharvest loss of banana occurs at different levels of the supply chain [9]. Using banana pulp in industrial processes provides a great opportunity for gluten-free product development and reduces postharvest loss [1].

The drying technique is one of the conventional methods for extending the storage time of fruits and vegetables without significant changes [6]. Fruit pulp drying is complicated due to the presence of low-weight molecules like fructose, glucose, sucrose, and organic acids, all of which make the drying process more complex [10]. With a high initial moisture content of 72 to 77% (wet basis) for bananas, the fruit is more likely to be damaged or decayed. In addition, bananas are a greatly perishable fruit with a relatively inadequate shelf life, making the distribution of fresh fruits to various locations quite challenging [11]. The dried powder of the fruits can be preserved for a longer period, making it available year-round to prepare different types of baked products [12]. Nutritional, functional, and sensorial attributes of the developed flours depend on the composition of the fruit cultivars and the drying conditions [13]. Freeze drying is a wonderful method of producing high-quality flour while preserving the original color, flavor, aroma, and excellent structural and functional attributes [14]. Freeze drying is the process of direct dehydration through the sublimation of frozen water from the materials [15]. Cabinet drying is the most popular and least expensive technique of drying, providing users with greater flexibility [16]. In cabinet drying, moisture is evaporated from the materials by circulating hot air throughout the drying chamber [17]. Microwave drying is the process by which the water molecules in the materials absorb microwave-emitted radiation and induces water evaporation by raising the temperature [18]. The forced air drying technique accelerates the drying rate of the materials by increasing the velocity of the heated air and decreasing the relative humidity of the drying chamber [19]. Because of its high mineral concentration, banana powder can be used as a dietary mineral supplement. It possesses high potassium, which is necessary for the heart's healthy operational function and blood pressure maintenance. Drying procedures are the only way to preserve essential micronutrients [20]. Banana powder is used in various meals, including cakes, breads, cookies, baby food, ice cream, flavored milk, and chocolates. As a result, they must be transformed into value-added items that retain their color, flavor, and nutrients while having a longer shelf life. It is necessary to apply appropriate processing technology to extend the shelf life of fruits due to their high perishability and enzymatic action of glycosidase and polyphenol oxidase. Among the most popular processing technologies, drying is popular and mainly used technology for that purpose [21].

Developing quality banana flour by proper drying method is the best alternative to reducing banana wastage during peak production and supplying low-cost raw materials for baking products, weaning and supplementary foods. Quality banana flour has good prospects in business opportunities and the development of rural agro-industries to produce value-added diversified processed products. The food processing industry favors high-quality flours with strong reconstitution properties and excellent sensory features at a reasonable price. The objectives of the study are to examine the effects of various drying methods on nutritional, functional, aesthetic quality, and microstructural changes of the developed banana flours from mostly grown banana cultivars.

## 2 Materials and methods

### 2.1 Experimental site and duration

The research was conducted in the analytical lab of the Agro Product Processing Technology department at Jashore University of Science and Technology in Jashore, Bangladesh, from July 2022 to December 2022. Two native popular banana fruit cultivars, Mehersagar and Sabri, were used in this study which was collected from the farmer's banana fruit orchard located at Churamonkati union of Jashore Sadar Upazila of Jashore District in the Division of Khulna, Bangladesh. The farmer is a commercial banana cultivar who collected the tissue-cultured banana plant from the genome center of the Horticulture division of Bangladesh Agricultural Research Institute (BARI), Jashore, Bangladesh. The orchard was located at the latitude and longitude 23°13′26.0″ N and 89°09′27.6″ E, respectively, and 11 m altitude from the sea level. During the study period, the average maximum and minimum temperatures were 37.1 °C (98.8 °F) and 14 °C (57.2 °F), respectively.

### 2.2 Experimental sample processing and study design

The experimental samples were harvested at the matured green stage and brought to the analytical laboratory of the Department of Agro Product Processing Technology, Jashore University of Science and Technology, Jashore. After sorting out the bruised, injured, and damaged, fresh banana fruits were stored in a closed chamber for 72 h at ambient temperature for complete ripening and color development. The experiment was followed by a Completely Randomized Design (CRD) with three replications. The experimental treatments were two native banana fruit cultivars viz. i. Mehersagar and ii. Sabri; and four drying methods were applied to develop quality banana flour viz. i. Freeze drying (FD), ii. Cabinet drying (CD), iii. Microwave oven drying (MOD), and iv. Forced air oven drying (FOD). Each cultivar's of banana fruits was cleaned individually under running water and the outer peel was removed with a sterilized sharp knife. Then, the samples were sliced at 5 mm thickness by hand slicer and dried based on the drying systems of freeze drying (Vertical Freeze Dryer BK-FD12P, China) for 48 h at −40 °C, cabinet drying (FP 240-UL, USA) for 10 h at 60 °C, Microwave oven drying (Samsung, CE138XAT, Thailand) for 1.5 h at 300 w, Forced air oven drying (Phoenix Instruments TIN-TF50S, Germany) for 12 h at 50 °C. The drying conditions of each treatment were optimized followed by the constant weight balance of the dried slices. The dried samples of each treatment were ground by a heavy-duty grinder and sieved through a 40 mesh size standard sieve. The obtained flour samples were sealed in a high-density polyethylene package perfectly and stored for future analysis.

### 2.3 Functional properties

#### 2.3.1 Water holding capacity (WHC) and oil absorption capacity (OAC)

The water holding capacity (WHC) and oil absorption capacity (OAC) of the obtained banana flour were determined following the centrifuge technique proposed by Rodrioguez-Ambrize et al. [22]. For WHC, the samples were weighed (0.5 g) accurately and taken into a centrifuge tube, along with 10 ml of distilled water. After stirring for 1 min with a vortex stirrer, kept at room temperature for 30 min and then centrifuged at 3000 rpm for 30 min. After centrifugation, the supernatant was then discarded, and the tube was turned upside down for 1 min. The procedure for oil absorption capacity was interrelated to WHC but distilled water was a substitute for 5 ml of commercial olive oil. equations (1), (2) were used to determine the WHC and OAC.

(1) $$Water\;holding\;capacity\;\left(g\;water/g\;dry\;sample\right)=\frac{Wet\;sample\;weight\;\left(g\right)-Dry\;sample\;weight\;\left(g\right)}{Dry\;sample\;weight\;\left(g\right)}$$

(2) $$Oil\;absorption\;capacity\;\left(g\;oil/g\;dry\;sample\right)=\frac{Oil\;absorbed\;sample\;weight\;\left(g\right)-Dry\;weight\;sample\;\left(g\right)}{Dry\;weight\;sample\;\left(g\right)}$$

#### 2.3.2 Swelling capacity (SC)

The swelling capacity (SC) was performed by the bed volume technique proposed by Dossou et al. [23] with some alterations. The sample of banana flour was taken up to 10 ml in a 100 ml graduated measuring cylinder, and added distilled water to adjust the 50 ml volume. The cylinder was covered and inverted for adequately mixed up. After 2 min, the cylinder was inverted again and kept for 30 min in stable condition. The sample volume was measured after 30 min and calculated with the following formula (3).

(3) $$Swelling\;capacity\;\left(g\;paste/g\;dry\;sample\right)=\frac{Swelling\;volume\;\left(ml\right)}{Weight\;of\;mucilage\;\left(g\right)}$$

#### 2.3.3 Bulk density

The bulk density was calculated using a minor alteration of the method described by Saifullah et al. [24]. A 10 ml graduated measuring glass cylinder was filled with 1 g of the sample weight to measure it. After that, the cylinder was gently shaken and pounded five times to flatten the top of the flour within. The bulk density was then estimated by dividing the sample mass by the volume of the measuring cylinder (4).

(4) $$Bulk\;density\;\left(g/ml\right)=\frac{Sample\;mass\;\left(g\right)}{Volume\;occupied\;by\;sample\;\left(ml\right)}$$

#### 2.3.4 Tapped density

The tapped density of the samples was determined using a slight change in the process outlined by Ozdikicierler et al. [25]. Putting 1 g of sample powder into a 10 ml graduated glass measuring cylinder, gently tapping the cylinder with a glass rod while resting it on a soft, stable surface until no volume change occurred, and recording the results, we computed the tapped density. The sample mass was divided by the tapped volume to calculate the tapped density (5).

(5) $$Tapped\;density\;\left(g/ml\right)=\frac{Mass\;of\;sample\;\left(g\right)}{Volume\;of\;sample\;after\;tapping\;\left(ml\right)}$$

#### 2.3.5 Carr Index

The sample's flowability is classified using the Car Index, which is the tapped density divided by the difference between the tapped and bulk densities. According to Dhakal et al. [26], the Car index also shows powder compressibility; a higher Car index value indicates poor flow ability and high compressibility. According to the approach proposed by Lebrun et al. [27], there are multiple Carr index ranges for categorizing the flow ability of a powder. The Car index is determined as follows (6):

(6) $$Car\;Index\;\left(\%\right)=\frac{Tapped\;density-Bulk\;density}{Tapped\;density}\times 100$$

#### 2.3.6 Hausner ratio

Carr's and Hausner's ratios were calculated based on the initial and final volumes of the flour but they do not specify the ease and speediness of consolidation, which was measured with the procedure described by Caliskan and NurDirim [28]. Some substances may compress fast while having a high index (which indicates limited flow). The relationship between Hausner's ratio and flow character was evaluated as per the standard chart [29]. The following equation (7) was used to determine the Hausner ratio.

(7) $$Hausner\;ratio=\frac{Tapped\;density}{Bulk\;density}$$

### 2.4 Physico-chemical properties

#### 2.4.1 Color parameters

The banana flour color was measured by the instrumental measurement in triplicate using a Chroma Meter (CR-400/410, KONICA MINOLTA, Japan). Before getting the reading, the chroma meter was accurately calibrated using a standard white reference tile. The samples were collected and placed on top of a Glass Light-Projection tube (CR-A33f, Konica Minolta, Japan) while the reading was obtained. According to the Chroma meter, the L axis represented lightness (If, L* = 0; black and L* = 100; white). The redness to greenness was displayed on the a-axis as (if, a*-value = +a*; redder and a*-value = -a*; greener). However, the b*-value showed a change in color from yellow to blue (if b*-value = +b*, yellower, and b*-value = - b*, bluer).

#### 2.4.2 Moisture content

In the experiment, moisture content was determined regarding the methods followed by AOAC [30]. At first, the weight of the Petri dish was recorded and tabulated. In the Petri dish, 5 g sample of each portion was taken. The Petri dish's and sample's weight were measured and recorded once again. The samples were then placed in Petri dish and kept in the oven at 105 °C for 6 h. Desiccators were used to bring the sample temperature to ambient after it had been heated. The weight of the Petri dish containing the sample was measured again and again until three consecutive weights became similar. The following formula (8) was then used to obtain the moisture percentages.

(8) $$Moisture\;content\;\left(\%\right)=\frac{Fresh\;weight\;of\;sample\;\left(g\right)-Dry\;weight\;of\;sample\;\left(g\right)}{Fresh\;weight\;of\;sample\;\left(g\right)}\times 100$$

#### 2.4.3 Ash content

The sample's ash amount was determined following the procedure explained by AOAC [30]. In a crucible, a 2 g sample was taken. The crucible was kept on a burner and heated over a low flame to fill it with all of the charred material. The crucible was then placed in a muffle furnace at 600 °C for 6 h. The crucible was then desiccated and weighed after cooling. To ensure the ashing process was completed, the crucible was reheated for 0.5 h and then weighed. This method was continued until the ash was practically whitish or grayish, and three consecutive weights were the same and calculated using formula (9).

(9) $$Ash\;content\;\left(\%\right)=\frac{weight\;of\;residue\;\left(g\right)}{Fresh\;sample\;weight\;\left(g\right)}\times 100$$

#### 2.4.4 pH and total soluble solids

The Hanna Instrument HI-2211 pH meter was used to measure the sample's pH value. To be precise, the 8% (w/v) flour suspension was swirled for 10 min and then left to stand for 5 min. The suspension was spun in a centrifuge, the rest was collected, and a reading was taken using the procedure given by Alkarkhi et al. [31]. Using a digital refractometer (HI 96801 refractometer, China), the suspension's total soluble solids (TSS) content was assessed using the method explained by Salvador et al. [32].

#### 2.4.5 Crude fat

The soxhlet extraction procedure recommended by AOAC [30] was used to determine the samples' fat content. Accurately, 5 g powder samples were weighed and taken into a thimble. The thimble was set in the soxhlet extraction chamber. About 90 ml petroleum ether was taken in the round bottom flak, assembled with the extraction chamber, and then placed over the heating mantle. The temperature of the heating mantle was adjusted so that solvent drips from the condenser at the rate of 3 drops per second and continued the extraction for 8 h. The extraction flask was detached and placed in an oven at 80 °C and dried the contents until constant weight is obtained. Then cooled, the flask in a desiccator, and measured the weight of the flask. The quantity of fat was estimated in percentage using the following formulae (10).

(10) $$Crude\;fat\;\left(\%\right)=\frac{Weight\;of\;flask\;with\;extracted\;fat\;\left(g\right)-Weight\;of\;empty\;flask\;\left(g\right)}{Weight\;of\;sample\;\left(g\right)}\times 100$$

#### 2.4.6 Crude fiber

The crude fiber content was determined following the method described by the AOAC method [30]. The flour sample (2 g) was measured and transferred to a 600 ml glass beaker. Sulfuric acid of 200 ml was added with bumping granules to the beaker near boiling. Then the samples were boiled for 30 min by rotating periodically. Afterwards, the sample was filtered through a reflux valve attached to the rubber topper. After filtration, the residue was washed with warm water and 12.5% sodium hydroxide up to dryness. The residue was taken in the crucible, dried for 2 h at 130 °C, and cooled in a desiccator. The samples were ashed at 550 °C, and cooled, the final weight was measured, and the crude fiber was calculated as a percentage by equation (11).

(11) $$Crude\;fiber\;\left(\%\right)=\frac{Weight\;of\;oven\;dried\;sample\;\left(g\right)-Weight\;of\;sample\;after\;ashened\;\left(g\right)}{Weight\;of\;sample\;\left(g\right)}\times 100$$

#### 2.4.7 Crude protein

According to Zhang et al. [33] the Micro-Kjeldahl method was used to determine the total nitrogen content. In order to determine the protein concentration, nitrogen was multiplied by a factor of 6.25. When sulfuric acid and nitrogen in the sample were digested at 380 °C in the presence of a catalyst combination, the nitrogen was transformed to ammonium sulphate. The nitrogen and protein percentages were estimated using the following (12), (13), and the ammonia was generated by distilling the digest with sodium hydroxide (NaOH) solution enchanted by boric acid.

(12) $$Nitrogen\;\left(N_{2}\right)\%=\frac{\left(V_{1}-V_{2}\right)\times N\times 0.014}{Weight\;of\;sample\;\left(g\right)}\times 100$$

Where, V_1_ = ml of HCl for sample; V_2_ = ml of HCl for Blank and N = Normality of HCl

(13) Proteincontent(%)=Nitrogen(%)×Conversionfactor(6.25)

#### 2.4.8 Determination of carbohydrates

According to Rangana [34], the total carbohydrate content was calculated by subtracting the percentages of protein, fat, ash, and moisture from hundred. The calculation was followed by formula (14).

(14) Carbohydrates(%)=100−[Moisture(%)+Crudefiber(%)+Ash(%)+Fat(%)+Protein(%)]

#### 2.4.9 Determination of energy content

The energy content of the powdered samples was determined by calculating the energy obtained from the amount of protein, fat, and carbohydrate of the respective sample. The energy content was calculated by the following formula (15).

(15) Energy=(Protein×4)+(Fat×9)+(Carbohydrate×4)kcal/100g

### 2.5 Bioactive compounds and antioxidant activity

#### 2.5.1 Sample extraction

According to the Ding et al. [35] method, the extracts were made using flour samples from different banana cultivars. In the extraction process, 1 g dried powder sample was weighed, and 10 ml 95% ethanol solvent was added and homogenized for 5 min using an Ultra-Turrax homogenizer. Following a 15 min at room temperature blending time, the homogenates underwent for 5 min, 3000 rpm centrifugation. Repetition of the extraction process was done after collecting the supernatant. The obtained extracts were stored at −20 °C as a stock solution for further use.

#### 2.5.2 Total flavonoid content

The method that was described by Chhetry et al. [36] used with minor modifications for determining the total flavonoid content. Different quercetin solution concentrations were prepared from stock solution (1 mg/ml) using ethanol solvent. At first, prepared 1 mg/ml concentration of the ethanolic extract. Then 1 ml of extract was dissolved with 4 ml of distilled water and 0.3 ml of 5% NaNO_2_. After 5 min resting time, 0.3 ml of 10% AlCl_3_ reagent solution was added and incubated for another 5 min. Then 2 ml of 1 M NaOH was added to the solution. Accordingly, a blank solution was also prepared without sample. All the reaction mixtures were incubated for 30 min at room temperature for a complete reaction. The absorbance was measured at 430 nm by a double beam Scientific UV–Vis Spectrophotometer when 30 min of incubation at room temperature. The total flavonoid content was calculated using the standard calibration curve for Quercetin.

#### 2.5.3 Total phenolic content

According to Wootton-Beard et al. [37], the modified Folin-Ciocalteu procedure was used to determine the total phenolic content of the flour samples. One (1) ml of each ethanolic extract of 1 mg/ml was blended with 5 ml of Folin-Ciocalteu reagent (1:10 v/v in distilled water) and 4 ml of 7.5% (v/v) sodium carbonate solution. Then the mixture was vortexed for 15 s for color development and left at 40 °C for 30 min. A double-beam Thermo Scientific UV–Vis Spectrophotometer was used to detect the absorbance at 765 nm. Instead of the sample, water was used to make the blank. A blank was used to compare a set of Gallic acid standard solutions. Total phenolic content was calculated as mg of Gallic acid equivalents by using the linear equation of a standard gallic acid calibration graph.

#### 2.5.4 DPPH scavenging activity

The method reported by Bagale et al. [38] the sample's DPPH free radical scavenging activity was determined with slight modification. Initially, the stock solution of 0.1 mM of DPPH, 1 mg/ml of ascorbic acid, and the test solution were prepared using ethanol. To conduct this test, 2 ml of ethanolic extract (1 mg/ml) solutions at different concentrations were added to the same amount of 0.1 mM DPPH solutions. The mixture was vigorously agitated for 15 s. The solutions were left to stand for 30 min at room temperature in a dark area for the reaction to occur. Using a twin beam Scientific UV–Vis Spectrophotometer, absorbance was measured after 30 min against a blank at 517 nm (T60, UV–Vis Spectrophotometer). Ethanol and ascorbic acid were chosen as blank and positive controls, respectively. The free radical scavenging activity was expressed as an inhibition percentage and was calculated using the following formula (16).

(16) $$Inhibition\;\left(\%\right)=\left(\frac{Ac-As}{Ac}\right)\times 100$$

Where, Ac = Control absorbance and As = Sample absorbance.

### 2.6 Sensory attributes

The consumer acceptability of the developed banana flours was assessed by a 9-point hedonic scale [39]. Before sensory evaluation, we confirmed that the experiment followed the established ethical guidelines. Consent from all participants was taken before conducting the experiment. According to Lawless and Heymann [40], sensory analysis employs groups ranging from 50 to 150 untrained subjects for product development. A blind sensory evaluation sheet was provided to fifty participants individually, and they were asked to give the sensory score. The sensory score was given based on the quality attributes of appearance, fineness, taste, flavor, color, and overall acceptability on a 1-9-point scale, where 9 = Like extremely, 8 = like very much, 7 = Like moderately, 6 = Like slightly, 5 = Neither like or dislike 4 = Dislike slightly, 3 = Dislike moderately, 2 = Dislike very much and 1 = Dislike extremely.

### 2.7 Scanning electron microscopy (SEM) analysis

Microstructures of the samples were analyzed by scanning electron microscopy (SEM) using ZEISS 1550VP Field Emission SEM with Oxford EDS and HKL EBSD at the genome center of Jashore University of Science and Technology, Jashore, Bangladesh. The SEM analysis was performed by computer meticulous field emission scanning microscope (SEM) equipped with an energy-dispersive X-ray system. The dried samples fragment were mounted on the aluminum stub and fixed onto the specimen holder. The sample's surface was gold coated very thinly in a coating chamber with the help of N_2_ gas to make the samples conductive. After then, the coated samples were positioned in the SEM-EDX chamber. The working condition was set according to the method described by Hossain et al. [41], at an accelerated voltage where electron high tension (EHT) was 5.00 kV, working distance (WD) 7.3–8.2 mm, and the high-resolution image of the microstructure was captured at 5.00 KX magnificence of 5 μm particle size.

### 2.8 Statistical analysis

For a better explanation, the experimental data of all the parameters were measured and subjected to statistical analysis. All data were accomplished in triplicate (n = 3) in number. The results were stated as the mean ± standard deviation, and the significance was analyzed by the analysis of variance (ANOVA) using Statistical Package for Social Sciences (SPSS) software 17.0 (IBM INC., New York).

## 3 Results and discussion

### 3.1 Functional properties

#### 3.1.1 Water holding capacity

The water holding capacity of flour is a hydration parameter that assesses the powder's ability to absorb water and attain the correct consistency [17]. As demonstrated in Table 1, the water holding capacity of the different banana cultivars flour prepared by various drying methods ranged from 0.93 ± 0.06 to 2.74 ± 0.06 g water/g dry sample. Among the treatments, the FD-Mehersagar cultivar showed the highest water holding capacity and FOD-Mehersagar was the lowest. In comparison, the FOD-Mehersagar cultivar of all treatments showed lower water holding capacity than FOD-Sabri. There were considerable changes in the water holding capacity of dried banana flour from cultivar to cultivar and drying method to drying method. According to Waliszewski et al. [42] water holding capacity relates to the state of the physical form of starch, dietary fiber, and protein content of the flour. According to Adebowale et al. [43], the structural condition of the starch polymer regulates the ability of water holding capacity. The smaller granular structure of starch polymer in freeze-dried banana powder offers a greater surface of hydration, increasing the water holding capacity. Also, the maximum protein retention in the freeze-dried powder results in greater water holding capacity and swelling power that facilitates the preparation of aqueous food formulations [44].

**Table 1:** Functional properties of the developed flours of the banana cultivars as influenced by different drying methods.

Banana cultivars	Drying methods	Water Holding Capacity (g water/g dry sample)	Oil Absorption Capacity (g oil/g dry sample)	Swelling Capacity (g paste\g dry sample)	Bulk Density (g/ml)	Tapped Density (g/ml)	Carr's Index (%)	Hausner Ratio
Mehersagar	FD	2.74 ± 0.06^a^	2.22 ± 0.10^a^	4.88 ± 0.01^c^	0.57 ± 0.02^de^	0.66 ± 0.02^de^	13.58 ± 0.43^a^	1.16 ± 0.00^a^
	CD	2.18 ± 0.11^c^	1.07 ± 0.06^de^	4.62 ± 0.02^f^	0.64 ± 0.02^c^	0.74 ± 0.03^c^	13.12 ± 0.42^ab^	1.16 ± 0.00^a^
	MOD	2.38 ± 0.013^b^	0.94 ± 0.05^ef^	4.67 ± 0.02^e^	0.81 ± 0.02^a^	0.92 ± 0.02^a^	11.27 ± 0.38^d^	1.13 ± 0.05^c^
	FOD	0.93 ± 0.06^f^	1.10 ± 0.08^d^	4.74 ± 0.04^d^	0.73 ± 0.03^b^	0.83 ± 0.03^b^	12.06 ± 0.44^cd^	1.14 ± 0.00^bc^
Sabri	FD	1.33 ± 0.07^d^	1.28 ± 0.09^c^	5.05 ± 0.03^a^	0.59 ± 0.02^d^	0.68 ± 0.02^d^	13.65 ± 0.42^a^	1.16 ± 0.00^a^
	CD	1.11 ± 0.06^e^	0.92 ± 0.04^f^	4.93 ± 0.03^b^	0.54 ± 0.04^e^	0.62 ± 0.04^e^	12.36 ± 0.46^bc^	1.15 ± 0.01^ab^
	MOD	2.28 ± 0.12^bc^	0.87 ± 0.06^f^	4.77 ± 0.01^d^	0.81 ± 0.03^a^	0.93 ± 0.03^a^	12.85 ± 0.86^abc^	1.15 ± 0.01^ab^
	FOD	1.30 ± 0.08^d^	1.83 ± 0.10^b^	4.92 ± 0.04^bc^	0.74 ± 0.02^b^	0.82 ± 0.02^b^	9.38 ± 0.47^e^	1.11 ± 0.05^d^

Here, values are means ± standard deviation (n = 3); Means with the same letter in a column are not statistically different from each other (p < 0.05); FD = Freeze Drying; CD = Cabinet Drying; MOD = Microwave Oven Drying; FOD = Forced Air Oven Drying.

#### 3.1.2 Oil absorption capacity

The oil absorption of produced banana flours from various cultivars and drying techniques was significant (Table 1). In comparison, FD-Mehersagar cultivar flour showed the highest oil absorption capacity (2.22 ± 0.10 g oil/g dry sample) while the flour of Sabri cultivar of microwave oven dried was found the lowest oil absorption capacity (0.87 ± 0.06 g oil/g dry sample). According to Rodrguez-Ambriz et al. [22], oil absorption capacity is related to the starch hydrophilic character contained in flour, which could be present in some proportion in both ripe and unripe banana flour. The greater surface polarity of the granules of food stuffs increases the oil absorption ability through the lyophilic colloidal process [45]. Hydrophilic chains in the amino acid molecules enhanced the oil absorption potentiality of the freeze-dried banana flour [46]. As freeze drying operates under low temperatures and high vacuum preventing the collapse of protein molecules and retaining the emulsifying capacity [47]. Flour's high oil absorption capacity indicates that it will be helpful in food preparations, giving better oil mixing properties to the baked product [48].

#### 3.1.3 Swelling capacity

The swelling capacity of the flour is the measure of its ability to swell by absorbing water by the starch granules. In Table 1, the swelling capacity was found highest in the freeze-dried flour of the Sabri cultivar 5.05 ± 0.03 g paste/g dry sample) and lowest in the cabinet dried Mehersagar cultivar flour 4.62 ± 0.02 g paste/g dry sample). Falodun et al. [49] discovered a substantial difference between freeze drying and cabinet drying, where freeze drying is more efficient than cabinet and sun drying. The swelling capacity of flour is affected by particle size, species diversity, and processing method or unit operations [50]. The consistency and cohesiveness of the microstructural network of uniform and small granular particle size of the freeze-dried powder with a larger amount of carbohydrates and protein, allows absorbing more water and expansion in water than coarse particles [46]. The maximum water holding capacity increases the swelling capacity of flour with low solubility, where water adsorption occurs without protein dissolutions [51]. High swelling power denotes excellency of flour quality as it is prone to give a rigid gel structure through water retention, which facilitates better texture with a high volume of premium grades baked products [52].

#### 3.1.4 Bulk density

Bulk density is also called volumetric density, which measures the mass of particles per unit volume. The microwave treated flour of both Mehersagar and Sabri cultivars had the maximum bulk density (0.81 ± 0.02 g/ml), while cabinet dried flour of the Sabri cultivar had the lowest (0.54 ± 0.04 g/ml). In comparison, Microwave oven drying presented the better bulk density among the treatments, which is related to the result obtained by Asif-Ul-Alam et al. [53]. Microwave dried powder possesses the intermolecular void volume due to spatial arrangements of granular particle that gives greater flowability to the powder. Compared to other drying methods, the microwave treatment gives the lowest porosity and highest bulk density volumes of the flour [54]. The higher bulk density facilitates the packaging process preventing particle aggregation [55]. According to Fegbemi [56], the flour produced had a bulk density ranging from 0.43 to 0.63 g/ml, which is in agreement with the results of this study.

#### 3.1.5 Tapped density

Tapped density of the flour from different banana cultivars of various drying methods was found to be significantly different. According to Table 1, the tapped density among the treated flour was ranged from 0.62 ± 0.04 to 0.93 ± 0.03 g/ml. In comparison, microwave oven dried flours of both Mehersagar and Sabri cultivars results in a greater value of tapped density. This is due to factors like granular size, solid density, geometry, and surface properties of the flour material which was also reported by Verma et al. [57]. Freeze-dried flour particles readily compact and collapse due to lower bulk density during tapping, occupying smaller volumes demonstrating low tapped density [58].

#### 3.1.6 Carr's Index and Hausner ratio

The flour samples' flowability and cohesiveness were described by the Carr Index and Hausner Ratio, respectively. The data in Table 1, showed the Carr's Index and Hausner Ratio values of flour samples with varying drying methods. The results revealed a considerable difference in all the samples' flowability and cohesiveness features. The flowability and Car Index qualities of both Sabri and Mehersagor cultivars revealed good quality with the Carrs Index and flowability relationship ranging from 9.38 ± 0.47 to 13.65 ± 0.42. The result is similar to the observation obtained by Koç et al. [59] that small particle size processed powder has poor flow properties. All the treated flours showed good cohesiveness with the range of Hausner ratio from 1.13 ± 0.05 to 1.16 ± 0.00 with the least significant differences. According to Bala et al. [60], the decreased powder size increases the Hausner ratio due to the van der Waals and the inter-particle forces of the smaller granular particles with greater cohesive. The range of Housner ratio in between 1.00 and 1.11 indicates the excellent flowability of flour, whereas 1.12 to 1.18 indicates good [29]. It can be stated that the flour prepared from each drying technique gives good flowability than excellent according to the results of our study.

### 3.2 Physico-chemical properties

#### 3.2.1 Color parameters

**Fig. 1:**
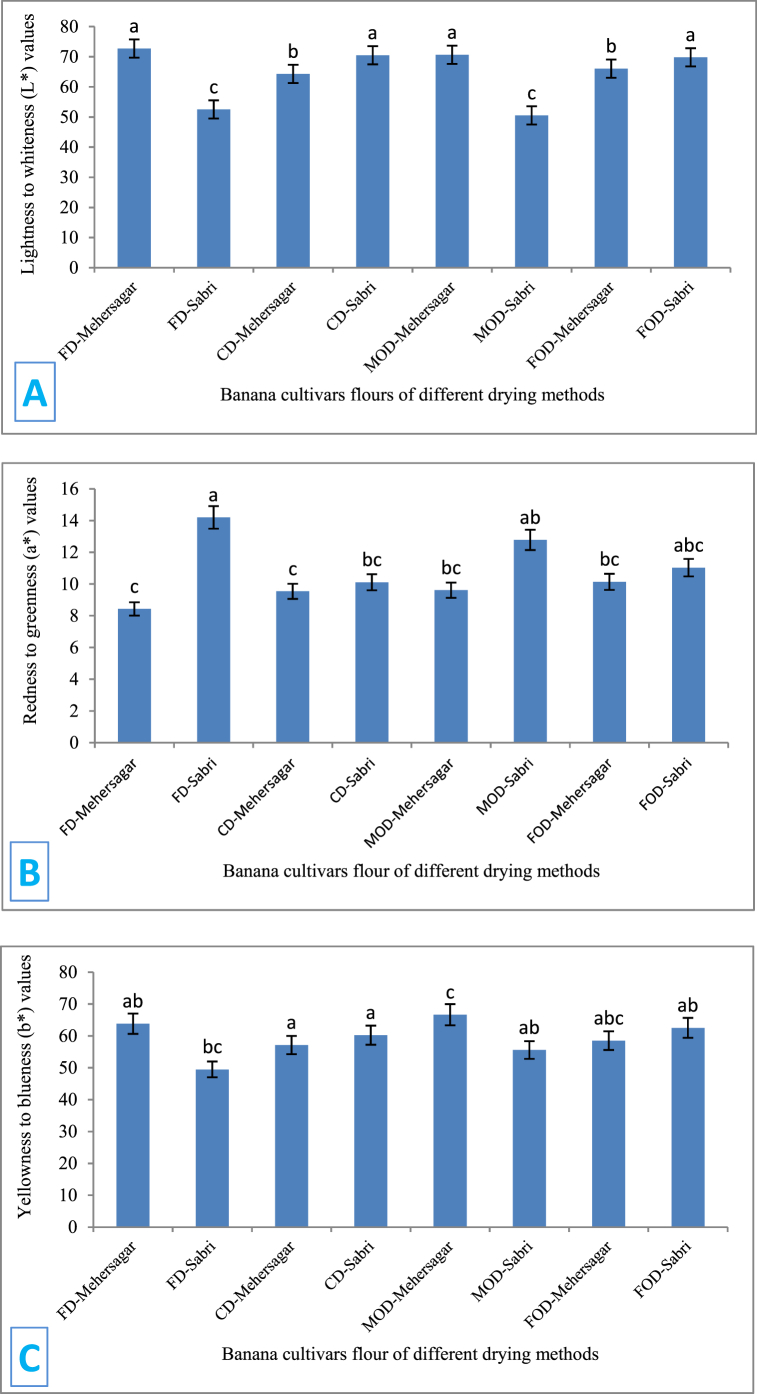
The color parameters of the banana cultivars flour with different drying methods indicating A. is lightness (L*), B. is redness to greenness (a*) and C. is yellowness to blueness (b*). The lettering above each bar represents the statistical differences of post-hoc Tukey's testing. (For interpretation of the references to color in this figure legend, the reader is referred to the Web version of this article.)

Color is an important quality parameter for dried materials. Thermal treatments usually alter the natural color of products, particularly fruits, and vegetables, which include a high proportion of water, carbohydrates, proteins, and lipid portions. Fig. 1(A-C), depicts the influence of various drying processes on the color (L*, a*, and b* values) of banana flours (Mehersagar and Sabri). The results showed no significant difference in the same treatment for both banana cultivars. According to Fig. 1 (A), the highest L* value was found in FD-Mehersagar (72.21 ± 1.05) and lowest in MOD-Sabri (50.51 ± 1.49). There was no significant difference in the CD-Sabri, MOD-Mehersagar, and FOD-Sabri treatments. Data demonstrated in Fig. 1 (B), the a* value ranged from 8.43 ± 2.07 to 14.2 ± 1.71. FD, MOD, and FOD treatments showed significantly similar results for both cultivars. The a* value of CD-Mehersagar (14.2 ± 1.71) was significantly similar to CD-Sabri but significantly different from the rest of the treatments for all the cultivars. The b* value, according to Fig. 1 (C), the highest value was found in FOD-Mehersagar (30.25 ± 1.33), which was not significantly different from MOD-Mehersagar (30.09 ± 0.71), FOD-Sabri (29.08 ± 1.16) but significantly different from rest of the treatments. According to the literature, freeze drying produces drier items with a greater degree of brightness than standard hot air drying [61]. The low drying temperature of freeze drying inhibits the Maillard reaction, caramelization, and the deterioration of color pigments preserving the natural color [62].

#### 3.2.2 Moisture content

Table 2, provided information on the moisture content of banana flours (Mehersagar and Sabri) processed using different drying methods. Mehersagar and Sabri cultivar flour samples differed greatly depending on the drying process. The moisture percentage of the samples ranged from 3.96 ± 0.09% to 7.74 ± 0.13%, with FOD-Sabri having the greatest moisture content and FD-Mehersagar having the lowest. On the drying procedures, the moisture content of both banana (Mehersagar and Sabri) flours showed the ascending order FD < CD < MOD < FOD. According to the findings of Sahoo et al. [63], the moisture content of the dried flour was lower than 6.92%. All treated flour obtained in this investigation had a moisture level of less than 10%, which is suitable for extended shelflife [64]. Because of the decreased potential for microbial growth and superior stability against physical and chemical reactions, the lower water content in all samples indicated improved stability and longer shelf life.

**Table 2:** Physico-chemical properties of the developed flours of the banana cultivars as influenced by different drying methods.

Banana cultivars	Drying methods	Moisture Content (%)	Ash (%)	TSS (°Brix)	pH	Crude Fat (%)	Crude Fiber (%)	Crude Protein (%)	Carbohydrate (%)	Energy (Kcal/100g)
Mehersagar	FD	3.96 ± 0.09^f^	3.54 ± 0.01^a^	5.91 ± 0.15^b^	4.50 ± 0.14^d^	0.21 ± 0.01^cd^	0.25 ± 0.02^g^	3.77 ± 0.03^bc^	88.26 ± 0.15^a^	370.02 ± 0.39^a^
	CD	6.26 ± 0.09^d^	3.17 ± 0.03^c^	4.37 ± 0.03^d^	5.20 ± 0.15^b^	0.34 ± 0.04^a^	0.93 ± 0.01^d^	3.71 ± 0.03^c^	85.60 ± 0.19^d^	360.27 ± 0.28^d^
	MOD	6.35 ± 0.06^cd^	2.44 ± 0.02^g^	3.12 ± 0.01^f^	5.72 ± 0.11^a^	0.11 ± 0.01^e^	0.64 ± 0.03^f^	2.72 ± 0.07^d^	87.73 ± 0.13^b^	362.82 ± 0.17^c^
	FOD	7.64 ± 0.12^a^	2.72 ± 0.04^e^	4.32 ± 0.06^d^	5.22 ± 0.07^b^	0.16 ± 0.03^de^	1.04 ± 0.02^b^	3.67 ± 0.05^c^	84.77 ± 0.24^e^	355.23 ± 0.51^f^
Sabri	FD	4.55 ± 0.07^e^	3.56 ± 0.02^a^	6.27 ± 0.09^a^	4.75 ± 0.16^c^	0.27 ± 0.01^b^	0.28 ± 0.02^g^	3.93 ± 0.06^a^	87.41 ± 0.17^b^	367.78 ± 0.35^b^
	CD	6.53 ± 0.13^c^	3.30 ± 0.02^b^	4.27 ± 0.04^d^	5.36 ± 0.13^b^	0.36 ± 0.04^a^	0.97 ± 0.02^c^	3.86 ± 0.05^ab^	84.98 ± 0.25^e^	358.57 ± 0.47^e^
	MOD	7.19 ± 0.16^b^	2.54 ± 0.01^f^	3.53 ± 0.07^e^	5.72 ± 0.05^a^	0.14 ± 0.01^e^	0.71 ± 0.01^e^	2.85 ± 0.16^d^	86.56 ± 0.34^c^	359.00 ± 0.60^e^
	FOD	7.74 ± 0.13^a^	2.79 ± 0.07^d^	5.15 ± 0.07^c^	5.37 ± 0.06^b^	0.25 ± 0.06^bc^	1.22 ± 0.03^a^	3.85 ± 0.11^ab^	84.15 ± 0.39^f^	354.25 ± 0.57^g^

Here, values are means ± standard deviation (n = 3); Means with the same letter in a column are not statistically different from each other (p < 0.05); FD = Freeze Drying; CD = Cabinet Drying; MOD = Microwave Oven Drying; FOD = Forced Air Oven Drying.

#### 3.2.3 Ash content

In the four drying methods, the ash content of two banana varieties was found to be highest in the Sabri variety owing to FD treatment (3.56 ± 0.02%) but not substantially different from Mehersagar due to the same treatment (3.54 ± 0.01%) (Table 2). MOD treatment resulted in the lowest ash content (2.54 ± 0.01%) in the Sabri variety. A noticeable difference was also observed in both types due to CD and FOD treatments. Freeze-dried (FD) samples had the greatest ash concentration among the other samples. On the drying procedures, the ash content of both banana flours (Mehersagar and Sabri) presented the order FD > CD > FOD > MOD. Furthermore, the samples had a considerable rise in ash content after drying due to water removal, which enhanced the nutritional concentration. Furthermore, the higher ash concentration after drying might be explained by the low volatility of minerals, which is not eliminated by heating. The ash content of food shows the total amount of minerals present and the presence of toxic metals such as lead [65]. For that reason, in some cases, there are limitations of maximum ash content in the food components [66].

#### 3.2.4 Total soluble solids (TSS)

TSS content in Table 2, revealed considerable variations among the treatments. Under the MOD treatments, the flour component of the cultivar Sabri flour of freeze-dried showed the highest TSS 6.27 °Brix, whereas the cultivar Mehersagar showed the lowest 3.12 °Brix. In comparison, the MOD treatment in both cultivars had the lowest TSS content. Golder et al. [67] found a higher total soluble solid in Sabri cultivar than native cultivar Amritsagar. The average TSS of Mehersagar banana flour was marginally lower than that of Sabri banana flour, indicating that Sabri banana flour contained more sugar. In ripe fruits, insoluble starch is converted into sucrose, increasing the total soluble solids. Bugaud et al. [68] also stated that there is a correlation between TSS and sucrose content in bananas. Starch content decreases from 70 to 80% in the pre–climacteric phase to less than 1% towards the end of the climacteric period [33], whereas sugars, primarily sucrose, increase to more than 10% of the fresh weight of the fruit.

#### 3.2.5 pH

The pH of a food is an excellent predictor of its potential to develop microorganisms. According to Table 2, the highest and most similar pH (5.72 ± 0.11 and 5.72 ± 0.05) were obtained in MOD-Mehersagar and MOD-Sabri flour samples, respectively. The FD-Mehersagar cultivar has the lowest rating (4.50 ± 0.14). During drying procedures, the pH of both banana flours (Mehersagar and Sabri) follows the ascending order FD < CD > FOD > MOD. According to Verma et al. [57], rapid drying eliminates water molecules by trapping acid molecules, which increases acidity. Kabeer et al. [17] stated that freeze-dried ripe banana flour was highly acidic compared to the cabinet dried and spray dried banana flour, which is in agreement with our result.

#### 3.2.6 Crude fat

Bananas of two different cultivars were put through four different processes, and their crude fat content ranged from 0.11 ± 0.01% to 0.36 ± 0.04%. The crude fat of CD in the Sabri variety (0.36 ± 0.04%) was not significantly different from CD in the Mehersagar variety (0.34 ± 0.04%) which was higher than the rest of the drying methods of both varieties. According to the findings, microwave oven-dried banana flour has the lowest fat level of any treatments. This is due to the breaking of fat globules during microwave drying at high temperatures. The presence of fat content in the freeze-dried flour samples could improve their flavor retention capacity while rendering them suitable for certain fat-soluble vitamins [49].

#### 3.2.7 Crude fiber

According to Table 2, substantial variations in crude fiber content were observed in different drying techniques for both banana cultivars. The FOD-Sabri variety contained the most crude fiber (1.22 ± 0.03%), whereas FD-Mehersagar contained the least (0.25 ± 0.02%). According to Falodun et al. [49], the percentage of crude fiber for cabinet dried cardaba banana flour was 0.84, which is relevant to our findings. Although there were significant variations between them due to the variety difference, the forced air oven drying method retained the crudest fiber content for both types. On the drying procedures, the crude fiber of both banana flours (Mehersagar and Sabri) follows the declining order of FOD > CD > MOD > FD.

#### 3.2.8 Crude protein

The Sabri variety had the highest crude protein content, with less significant differences in other treatments ranging from 3.85 ± 0.11% to 3.93 ± 0.06%, except for the MOD treatment (2.85 ± 0.16%) (Table 2). Mehersagar and Sabri banana flours were dried using four distinct techniques, each resulting in a particular amount of crude protein. The highest crude protein (3.93 ± 0.06%) was detected in Sabri flour dehydrated by FD, whereas the least (2.72 ± 0.07%) was found in MOD-Mehersagar flour. The MOD treatment of both fruits maintained considerably less crude protein than the other techniques and was found to have the lowest crude protein content (2.72 ± 0.07% and 2.85 ± 0.16%). The crude protein content of freeze-dried banana flour recorded in the literature was 1.45% in Sagor banana [69] and 1.07% in Nendran banana [17]. The crude protein of both banana (Mehersagar and Sabri) flours on the drying methods follow the descending order FD > CD > FOD > MOD. The FD method retained the highest quantity of crude protein for both varieties of bananas in this study. Freeze drying utilizes low pressure and temperature, keeping the banana's cellular structure unaltered and reducing protein denaturation [70].

#### 3.2.9 Carbohydrate content

The carbohydrate levels of both banana species exposed to four drying methods differed significantly.

Carbohydrate levels in the flour of FD-Mehersagar (88.26 ± 0.15%) were substantially greater than in the Sabri variety (87.41 ± 0.17%) of dry weight. FOD treatment resulted in the lowest carbohydrate content (84.77 ± 0.24%), but Mehersagar was substantially greater than the Sabri cultivar (84.15 ± 0.24%) of dry weight. Carbohydrate, ash, and fiber levels are inversely related to the amount of moisture lost due to dehydration. The carbohydrate content of both banana flours (Mehersagar and Sabri) on drying processes is listed in descending order of FOD > MOD > CD > FD. According to Falodun et al. [49], Freeze-dried cardaba banana flour retained the highest amount of carbohydrates than cabinet drying. The ripe banana flour exhibited a large amount of carbohydrates, corresponding with the scientific consensus that an elevated amount of sugars, starches, and dietary fibers [71].

#### 3.2.10 Energy content

According to Table 2, the energy value of banana varieties varies greatly. The highest quantity of energy content (370.02 ± 0.39 kcal/g of dry sample) was observed in FD-Mehersagar flour while lowest quantity (354.25 ± 0.57 kcal/g of dry sample) was in FOD-Sabri. The energy content of both banana (Mehersagar and Sabri) flours on the drying methods follows the descending order FD > MOD > CD > FOD. According to the observations, the moisture content of the dry samples has an inverse relationship with the energy content, but the TSS has a proportional relationship.

### 3.3 Determination of bioactive compounds and antioxidant activity

#### 3.3.1 Total flavonoid content

As shown in Table 3, both banana (Mehersagar and Sabri) flours have diverse flavonoid content. The experimental findings showed that, after being subjected to four different drying techniques, the flour of the two banana cultivars (Mehersagar and Sabri) significantly varied. The results showed that FD-Mehersagar had the highest total flavonoid content (34.34 ± 0.03 mg QE/100g dry weight), while MOD-Sabri had the lowest (21.44 ± 0.04 mg QE/100g dry weight). The maximum quantity was discovered in CD treatment for the Sabri variety was 30.85 ± 0.02 mg QE/100g dry weight; a similar result was found by Falodun et al. [49]. Direct exposures to high heat and light for a longer time destroy the enzymatic activity and block the flavonoid synthesis pathways, which is the main reason for lower flavonoid content in microwave oven dried banana flour [72]. The structure-activity relationship of flavonoids due to the presence of functional groups in their nuclear structure also regulates their antioxidant capacity, scavenging free radicals and chelating action [73].

**Table 3:** Bioactive compound of banana flour as influenced by different drying methods.

Banana Cultivars	Drying methods	Total Flavonoid content (mg QE/100g dry sample)	Total Phenolic Content (mg GAE/100 g dry sample)
Mehersagar	FD	34.34 ± 0.03^a^	71.46 ± 0.03^a^
	CD	32.94 ± 0.04^b^	67.24 ± 0.04^c^
	MOD	31.75 ± 0.04^c^	29.91 ± 0.01^g^
	FOD	29.74 ± 0.05^e^	43.06 ± 0.02^d^
Sabri	FD	28.27 ± 0.06^f^	40.76 ± 0.05^e^
	CD	30.85 ± 0.02^d^	70.54 ± 0.02^b^
	MOD	21.44 ± 0.04^h^	36.93 ± 0.02^f^
	FOD	26.53 ± 0.02^g^	40.75 ± 0.05^e^

Note: Values are means ± standard deviation (n = 3); Means with the same letter in a column are not statistically different from each other (p < 0.05); FD = Freeze Drying; CD Cabinet Drying; MOD = Microwave Oven Drying; FOD = Forced Air Oven Drying.

#### 3.3.2 Phenolic content

The total phenolic content in two banana varieties subjected to four different treatments ranged from 29.91 ± 0.01 to 71.46 ± 0.03 mg GAE/100 of dry samples (as shown in Table 3). The maximum phenolic content was observed in the FD treatment for Mehersagar (71.46 ± 0.03 mg GAE/100 g dry weight). The MOD-Mehersagar found the lowest phenolic contents (29.91 mg GAE/100g dry weight). In the drying process, the phenolic compounds are detached from the oxidative enzyme making structural collapse and undergoing degradation, which causes more release of the compounds [74]. The rupture of ether and ester covalent bonds occurs at high temperatures and prolonged heat exposure and causes the losses of total phenolic [75]. Accordingly, the phenolic compound content in banana cultivars varies considerably for the production region, farming methods, soil types, environmental conditions, maturity, growing season, and post-harvest processing techniques [76].

### 3.4 Antioxidant activity

#### 3.4.1 DPPH scavenging activity

**Fig. 2:**
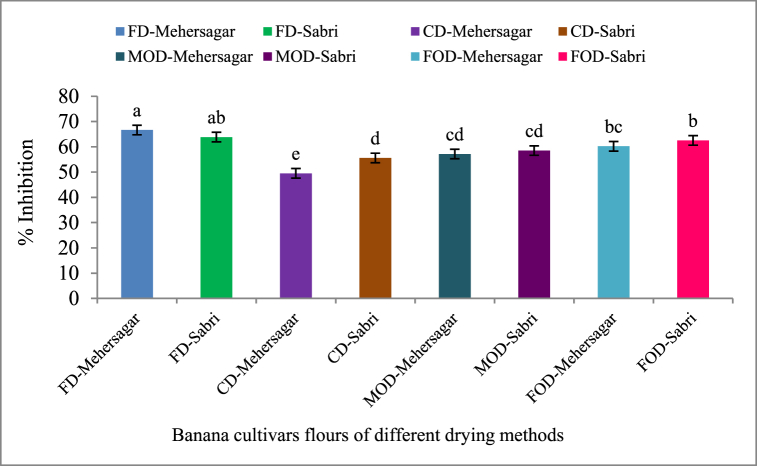
Effects of different drying techniques on DPPH scavenging activity of the banana flours. The lettering above each bar represents the statistical differences of post-hoc Tukey's testing at α = 0.05.

According to the findings in Fig. 2, there were significant (p < 0.05) differences in the total antioxidant activity of two banana cultivars (Mehersagar and Sabri) across all drying processes. The highest antioxidant activity (% Inhibition) was found in the FD-Mehersagar flour 66.65 ± 1.54%, and the lowest antioxidant activity (49.49 ± 1.65%) at the concentration of 1 mg/ml in the flour of Mehersagar dehydrated by cabinet drying method. In a comparison of antioxidant activity between both banana cultivars, the flour Mehersagar cultivar showed the highest inhibition % among the drying methods. According to Talha et al. [77], high processing temperature for flour preparation is responsible for the reduction of antioxidant activity. DPPH scavenging activity is negatively correlated with high temperature drying due to the denaturation of polyphenol oxidase and the reduced bioavailability of nutrients and other compounds involved in the antioxidant potentiality [78]. Also, the scavenging activity against DPPH radicals increased with the condensation of phenolic compounds.

### 3.5 Sensorial attributes

**Fig. 3:**
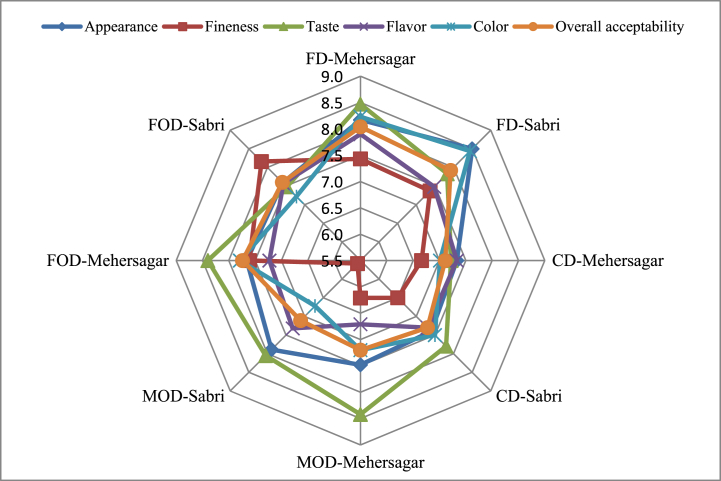
Drying effects on the sensorial attributes of the banana flours.

Different drying methods have impacts on the alteration of sensory properties of the dried fruit powders [78]. The average findings of the sensory evaluations were displayed as ratings on a nine-point hedonic scale in Fig. 3. The overall acceptability of FD, MOD, CD, and FOD for Mehersagar was found 8.04 ± 0.02, 7.20 ± 0.14, 7.32 ± 0.24 and 7.74 ± 0.04 respectively, and for Sabri it was 7.92 ± 0.17, 7.11 ± 0.11, 7.30 ± 0.09 and 7.60 ± 0.14 respectively. The highest score in the overall acceptability was found in the FD-Mehersagar cultivar, which was less significantly different from FD-Sabri. The lowest score in overall acceptability was found in CD and MOD treatments in both varieties. In cabinet and microwave oven, the dielectric constant generates uneven heating that causes an unpleasant appearance of the dried products. The FD banana flours (Mehersagar and Sabri) have retained the natural taste, flavor, and color than other dried samples. In freeze drying, low temperature helps keep the flour's original color and flavor. Similar findings were reported by Deepak et al. [79] who found that freeze-dried bananas ranked higher in terms of sensory attributes.

### 3.6 Scanning electron microscopy (SEM)

**Fig. 4:**
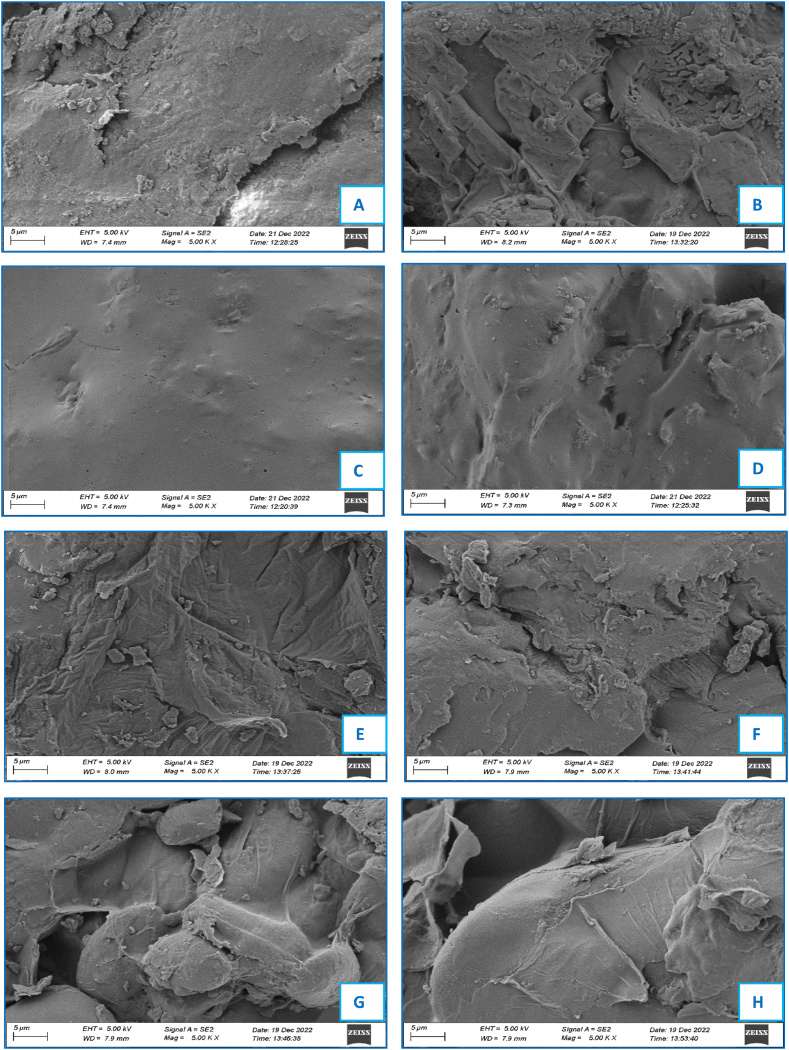
Microstructure of the banana flour granules treated with different drying methods where A). FD-Mehersagar; B). FD-Sabri; C). CD-Mehersagar; D). CD-Sabri; E). MOD-Mehersagar; F). MOD-Sabri; G). FOD-Mehersagar; H). FOD-Sabri. Scale bar = 5 μm at magnification of 5.00 KX, Working distance: 7.3–8.2 mm, Electron High Tension (EHT) voltage = 5.00 KV (FD = Freeze Drying; CD = Cabinet Drying; MOD = Microwave Oven Drying; FOD = Forced Air Oven Drying).

The effects on the microstructural changes of the developed flours of the banana cultivars by different drying methods were analyzed by scanning electron microscopy, and the captured high-resolution pictures depicted in Fig. 4(A-H). FD-Mehersagar flour exhibited discontinuous-like shiny granular rough surface (A), while the porous fluffy rough structure was found in FD-Sabri cultivar (B). The surface of the flour granules seems tiny, with a pore-like plane and dense consistency in CD-Mehersagar cultivar (C) and a smooth and irregular shape in CD-Sabri cultivar (D). In the microwave oven dried flour, the structure looks fibrous and fluffy in MOD-Mehersagar (E), while a rough structure with grits is revealed in MOD-Sabri (F) cultivar. Discontinuous and web-like plumy and spheroid structures were observed in FOD-Mehersagar (G), and spherical-like shiny and compact structures were found in FOD-Sabri (H). The microstructure showed significant variation in morphologies from cultivar to cultivar and drying methods. These microstructural conditions will assist in formulating nutraceuticals, pharmaceuticals, and cosmetic products. The morphology of the flour and its ability to contain water are correlated, as demonstrated by the SEM images of ripe banana flour [80].

## 4 Conclusion

The results of the research demonstrated that different drying techniques for banana flour preparation have profound impacts on the functional, physico-chemical, bioactive components, antioxidant properties, and sensory qualities. Due to having good functional properties and bioactive components, freeze-dried flour from Mehersagar and Sabri proved to be the most suitable drying technology for producing high quality flour. In comparison, freeze-dried Mehersagar cultivar flour performed best regarding sensory qualities while preserving its natural color, taste, and flavor. Forced air oven drying and cabinet drying are also recommended for producing flour with acceptable features; however, microwave oven drying is less satisfactory due to substantial changes in overall quality. It can be stated that the nutritional makeup of banana flour is regulated mainly through drying methods. The best-suited drying method will help to ensure the production of the best quality banana flour in perspective of functional and nutritional qualities. The high-quality banana flour may facilitate the preparation of high-value, nutrient-rich flour-based food products. Thus, converting perishable raw banana fruit into flour will help to increase market value, rendering easy storage and transportation with extended high-value shelflife and consumer availability.

## Ethics statement

These experiments were conducted according to established ethical guidelines, and informed consent obtained from the participants.

## Funding

This study did not receive specific grant from any agencies of the public or private sectors.

## Author contribution statement

Mahfujul Alam: Conceived and designed the experiments; Performed the experiments; Analyzed and interpreted the data; Wrote the paper.

Mrityunjoy Biswas: Contributed reagents, materials, analysis tools or data; Wrote the paper.

Mir Meahadi Hasan: Performed the experiments; Analyzed and interpreted the data; Wrote the paper.

Md. Faruk Hossain: Performed the experiments; Analyzed and interpreted the data.

Md. Ashrafuzzaman Zahid: Analyzed and interpreted the data; Contributed reagents, materials, analysis tools or data.

Md. Sajib Al Reza: Analyzed and interpreted the data; Wrote the paper.

Tarikul Islam: Conceived and designed the experiments; Performed the experiments; Wrote the paper.

## Data availability statement

Data will be made available on request.

## Declaration of competing interest

The authors declare that they have no known competing financial interests or personal relationships that could have appeared to influence the work reported in this paper.

## Figures and Tables

**Table:** 

General Information
Name (Optional):
Age:
Gender: □ Male □ Female
Date:
Assessment Instructions to participant
i) Please write the score in the score chart based on the hedonic scale (1–9) mentioned below the sensory attributes table.
ii) Please hold the sample in your mouth for minimum 5–10 s to judge properly.
iii) Please rinse your mouth with drinking water for each sample before the next sample judging.

**Table:** 

Banana cultivars	Drying methods	Quality attributes					
		Appearance	Fineness	Taste	Flavor	Color	Overall acceptability
A_1_	A_1_B_1_						
	A_1_B_2_						
	A_1_B_3_						
	A_1_B_4_						
A_2_	A_2_B_1_						
	A_2_B_2_						
	A_2_B_3_						
	A_2_B_4_						

Hedonic scale: 9 = Like extremely, 8 = like very much, 7 = Like moderately, 6 = Like slightly, 5 = Neither like or dislike, 4 = Dislike slightly, 3 = Dislike moderately, 2 = Dislike very much and 1 = Dislike extremely.

## Appendix 1 Sensory Evaluation Sheet

### Sensory Evaluation Sheet for

Quality Attributes Judgment of the Developed Banana Flour.
